# Efficient genetic transformation method for *Eucalyptus* genome editing

**DOI:** 10.1371/journal.pone.0252011

**Published:** 2021-05-24

**Authors:** Zechen Wang, Limei Li, Lejun Ouyang

**Affiliations:** 1 College of Biological and Food Engineering, Guangdong University of Petrochemical Technology, Maoming, Guangdong, China; 2 The Key Laboratory of Ecology and Biological Resources in Yarkand Oasis at Colleges & Universities under the Department of Education of Xinjiang Uygur Autonomous Region, Kashi University, Kashi City, Xinjiang Uygur Autonomous Region, China; National University of Kaohsiung, TAIWAN

## Abstract

Plantation forestry *of Eucalyptus urophylla × Eucalyptus grandis* supplies high-quality raw material for pulp, paper, wood, and energy and thereby reduces the pressures on native forests and their associated biodiversity. Nevertheless, owing to the heterozygosity of the *E*. *urophylla × E*. *grandis* genetic background, germplasm improvement by crossbreeding tends to be inefficient. As an alternative approach, genetic engineering of *Eucalyptus* can be used to effectively improve germplasm resources. From a strategic standpoint, increasing the plantation productivity and wood quality by transgenic technology has become increasingly important for forest industry. In this study, we established a fluorescence labelling method using CRISPR/Cas9 technology to obtain positive transformed progenies. The positive transformed progenies were easily obtained from the genetically modified population via fluorescence screening. This system can be used as a plant genome site-specific editing tool and may be useful for improving *Eucalyptus* genetic resources.

## Introduction

*Eucalyptus* is a fast growing genus, with high economic value, that is frequently planted over large areas worldwide (over 20 million hm^2^) [1]. *Eucalyptus* was introduced in China over 120 years ago. It remains a frequently-planted tree in 17 provinces (regions) of China, with a yearly timber yield of over 40 million m^3^. In addition, *Eucalyptus* provides more than 50% of the raw materials for pulpwood in China, making it an important afforestation tree species for maintaining timber safety in this country [2,3]. In addition, *Eucalyptus urophylla × E*. *grandis*, a hybrid species characterized by rapid growth and strong stress resistance, covers the largest cultivated area in China.

Over the recent years, as man-made *Eucalyptus* forests continue to expand, insect pests and plant diseases have been identified as leading factors limiting the development of man-made *Eucalyptus* forests [4]. The conventional chemical control has considerable side effects on the environment and ecological safety [5]. *Eucalyptus* breeding using modern biotechnologies such as transgenic technology can compensate for the shortcomings of conventional breeding technologies, shorten breeding cycles, and accelerate the breeding process in high-quality and highly resistant new cultivar [6]. In particular, investigating the functions of *Eucalyptus*-associated genes using CRISPR/Cas9 (Clustered Regularly Interspersed Short Palindromic Repeats/Cas9) technology is very important for improving and studying the functional genes in *Eucalyptus* [7]. One prerequisite for successful genetic engineering in *Eucalyptus* is establishing an efficient genetic transformation system. Genetic transformation of *Eucalyptus* usually uses non-single-cell calli as the explants, most of which are chimeras after transformation. The common method for screening resistance buds using antibiotics is extremely inefficient, and its accuracy is low. Currently, only a few studies have focused on finding an efficient genetic transformation system for *Eucalyptus* [4,8,9].

Successful A. tumefaciens- mediated transformation in most plants depends on the effectiveness of appropriate selection conditions. *mCherry* is a red fluorescent protein extracted from mushroom coral. *mCherry* is usually used to label and trace molecules and cellular components. Gao et al. [10] cloned *mCherry* close to the *At2S3* promoter expressed in the T_0_ seed coat of *Arabidopsis* to screen for positively transformed progenies, implementing efficient screening of transformants at an early stage of T_0_ plant transformation, thereby greatly improving work efficiency. This study used the efficient transformation system of *E*. *urophylla* × *E*. *grandis* established in our previous studies [4,11] to optimize parameters for genetic transformation of *E*. *urophylla* × *E*. *grandis* by orthogonal experiments [12]. *mCherry* was used as the fluorescence screening marker to examine positively transformed progenies, which laid a foundation for genetic improvement of CRISPR/Cas9 technology and studying relevant gene functions of *E*. *urophylla* × *E*. grandis. The selectable marker *mCherry* screening transformation method that can be used for future genome editing programs in *Eucalyptus*.

## Materials and methods

### Plant materials

*E*.*urophylla* × *E*. *grandis* DH32-29 aseptic seedlings were provided by the *Eucalyptus* Research and Development Center of the National Forestry Bureau.

### Strains and plasmids

*Agrobacterium tumefaciens* GV3102 and competent cells of *Escherichia coli* DH5α were purchased from Guangzhou Dingguo Biology. pHDE-*mCherry* and pHDE-Cas9 plasmids were a kind gift from Professor Zhao, University of California, San Diego.

### Primer design and synthesis

Primers were designed using Primer Premier 5 software based on the 35S and *mCherry* genetic sequences of the pHDE-*mCherry* template plasmid, and the sequences were analyzed using DNAStar software. All the primers were synthesized by Shengon Biotech (Shanghai) (Table 1).

**Table 1:** Primers sequences used in this study.

Primer name	Primer sequence
*35S*-F	TAATGCATTTTATGACTTGCAACATGGTGGAGCACGACAC
*35S*-R	CCAAGAGCTCCGTGTCCTCTCCAAATGAAA
*mCherry*-F	ATCGTTTTGCTCGAGAACCTGAGGGTACA
*mCherry*-R	GAATTCGTTGTCAATCAATTAATACGATAATTTATTTGAA
Cas9-F	CGAGGACATTGTGCTGAC
Cas9-R	GACGATATTCACTTGTGGC

### Construction of the recombinant plasmid pHDE/Cas9-35S-*mCherry*

**Fig 1:**
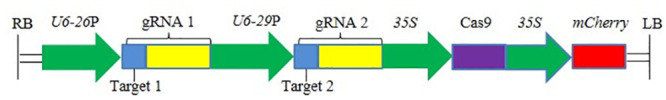
Schematic representation of recombinant plasmid pHDE-35S-*mCherry*. U6-26P and U6-29P are the promoters of two gRNAs, respectively. 35S is the promoters of Cas9, and 35S is the promoters driving of *mCherry* gene.

Plasmid pHDE-*mCherry* [13] containing *35S* or *mCherry* was used as the template to amplify *35S* or the fluorescence screening marker *mCherry* gene, respectively. PCR conditions was predenaturation at 94°C for 4 min, 35 cycles (denaturation at 94°C for 30 s, annealing at 56°C for 30 s, extension at 72°C for 45 s), total extension at 72°C for 2 min. The PCR products of *35S* and *mCherry* were used as the templates to perform fusion PCR amplification using the forward primer *35S-F* of promoter *35S* and reverse primer *mCherry*-R of *mCherry*; thus, a full-length segment of *35S* + *mCherry* was obtained. The obtained segment was tested by electrophoresis, then recollected from the gel (DNA gel extraction kit from Tiangen Biochemistry Company, Beijing). Homologous recombination was performed for this segment and the restriction endonuclease *EcoR*I-digested (Shengon Biotech, Shanghai) pHDE-Cas9 plasmid to form a recombinant plasmid pHDE/Cas9-*35S-mCherry*, and competent cells of *E*. *coli DH5*αwere transformed. The *E*. *coli* transformation protocol was according to the instructions of the kit (Shengon Biotech, Shanghai). After PCR verification of the recombinant plasmids, samples of positive recombinant plasmids were sent for sequencing. PCR conditions was predenaturation at 94°C for 4 min, 35 cycles (denaturation at 94°C for 30 s, annealing at 56°C for 30 s, extension at 72°C for 45 s), total extension at 72°C for 2 min. The components for vector construction are shown in Fig 1.

### Preparation and transformation of engineering bacterial solution

The recombinant cloning vector pHDE/Cas9-*35S*-*mCherry* was transferred into *A*. *tumefaciens* using the freeze-thaw method. When the concentration of bacterial solution OD_600_ was about 0.6, 1 mL bacterial solution was transferred into 50 mL fresh liquid culture medium without antibiotics and incubated at 28°C for 3~5 h. When OD_600_ of bacterial solution reached 0.3~0.6, the bacterial solution was transferred to a 2 mL centrifuge tube for centrifugation at 5000 rpm for 1 min. The bacteria at the bottom of the centrifuge tube were re-suspended using 1 mL of MS [14] liquid medium. The re-suspended bacterial solution was diluted 20 times with suspension culture medium—the same engineering bacterial solution used in the transformation.

### Orthogonal experimental design of the genetic transformation system

Five parameters affecting the transformation efficiency were determined based on a literature report [15], including pre-culture days (d), concentration of bacterial solution, pH value of infection solution, infection time, and co-culture days (d). The L_16_ (4^5^) orthogonal design (Table 2) was applied. There were 16 treatments with five duplicate culture dishes in each treatment, and 20 explants (stem segments (4–8 mm) were excised from aseptic seedlings) in each culture dish. The experiment was conducted based on the data in the Table 2. Pre-culturing, co-culturing, and selective induction of adventitious buds were performed for 30 d, then the induction rate of adventitious buds was calculated using the following formula: Induction rate of adventitious buds = (number of explants with bud differentiation/ number of total explants) ×100%.

**Table 2:** The orthogonal design table L_16_ of the genetic transformation system (4^5^).

Level of factors	Pre-culture days (day)	Concentration of bacterial solution(OD value) (OD_600_)	pH value of infection solution	Infection time (hour)	Co-culture days (day)
	A	B	C	D	E
1	2	0.3	5.2	1.0	2
2	4	0.4	5.4	1.5	4
3	6	0.5	5.6	2.0	6
4	8	0.6	5.8	2.5	8

The co-cultured explants were then transferred to 200 mg·L^-1^ of cephalosporins (Cef) selective differentiation medium, and the culture medium was replaced every 7~10 d. After transferring 2~3 times, Cef concentration in the culture medium was gradually reduced, and the buds that were growing poorly were eliminated. Adventitious buds with good growth were inoculated to the elongated medium. Adventitious buds that had elongated to 2~3 cm were cut off and planted in rooting medium to induce adventitious roots. Acclimatization and transplantation were performed when the seedlings had 5~10 stout adventitious roots about 5 cm long. The composition of the medium in each stage was referred to Ouyang [4].

### Visual screening of the transformed progenies

A fluorescence microscope was used to examine transformed progenies carrying *mCherry* (Excitation wavelength is 587 nm). The adventitious buds with red fluorescence were screened, isolated, and cultured separately for subsequent molecular identification.

### Detection of transgenic plants

Leaf DNA from *E*. *urophylla × E*. *grandis* intended for transformation was extracted using the CTAB method [16]. Cas9 identification primers were designed using Cas9 genetic sequences in the transformed plasmids. PCR identification of Cas9 was performed using the forward primer Cas9-F and reverse primer Cas9-R on plants with positive progenies identified during morphological screening (the adventitious buds with red fluorescence). PCR conditions was predenaturation at 94°C for 4 min, 35 cycles (denaturation at 94°C for 30 s, annealing at 56°C for 30 s, extension at 72°C for 45 s), total extension at 72°C for 2 min.

### Statistical analysis

The experimental design was completely randomized with five replicates of twenty explants. The values used for statistical analysis were the means obtained for each treatment. The treatment effects were analysed by ANOVA and means were compared by Duncan’s multiple range test (α = 0.05). All percentage data were subjected to arc-sine transformation prior to run ANOVA with SAS/STAT^®^ software [17].

## Results

### Construction of recombinant pHDE/Cas9-35S-*mCherry*

**Fig 2:**
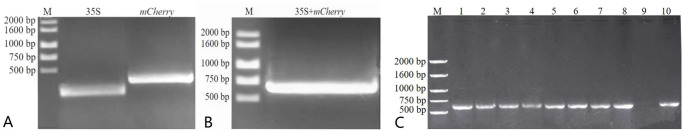
PCR product of Construction of recombinant pHDE/Cas9-35S-*mCherry*. A. PCR product of *mCherry* and 35S segment. B. Amplification of the “35S+*mCherry*” target. C. PCR product of recombinant plasmid pHDE-35S-*mCherry*. M: 2000bp DNA marker; 1–8: PCR of the recombinant plasmids; 9: Negative control; 10: Positive control.

*mCherry* (200 bp) and 35S promoter gene (450 bp) were amplified by PCR using pHDE-*mCherry* plasmid as the template (Fig 2A). The PCR products in the first cycle were recollected from the gel, then used as templates for overlapping PCR. Electrophoresis of the PCR products yielded a single band 500–750 bp long (Fig 2B), which is consistent with the size of “35S+*mCherry*”.

Consequently, amplified target segment “35S+*mCherry*” and *EcoR*I-digested pHDE-Cas9 plasmid were used to transform the competent cells after homologous recombination, which were then incubated in adventitious buds induction medium for transformation screening. Consequently, eight single colonies were randomly selected for liquid culturing. PCR detection of the bacterial solution was performed with the cultured bacterial solution as the template; 35S-F and *mCherry*-R were used as primers. The results showed 100% positive amplification (Fig 2C). The positive recombinant plasmids were then sent to Sangon Biotech (Guangzhou) for sequencing. The sequencing results were analyzed using DNAStar software. The results revealed that there were no abnormalities in the sequences corresponding to the peak, and no mutations at any base, which were consistent with the expected sequences. Therefore, recombinant vector pHDE-35S-*mcherry* was successfully constructed.

### Induction of calli after transformation

Table 3 shows that the following factors were significantly different: the viability of the calli formed by treatment with different pre-culture time, OD values of the bacterial solution, pH values of the infection solution, infection time, and co-culture time. Among these, the viability was high when using combinations 1 (A1B1C1D1E1), 7 (A2B3C4D1E2), and 8 (A2B4C3D2E1). Moreover, the viability of the calli affected the induction rate of adventitious buds to some extent. Therefore, preliminary analysis of the induction rate for the calli was conducive to subsequent experiments.

**Table 3:** Induction results of the calli using orthogonal experiments L_16_ (4^5^).

Treatment	Column number and effect					Explant viability		
	1	2	3	4	5			
	A	B	C	D	E	Very good	Good	No
1	1	1	1	1	1	89.87%	6.32%	3.70%
2	1	2	2	2	2	28.97%	38.31%	32.70%
3	1	3	3	3	3	71.74%	18.48%	8.41%
4	1	4	4	4	4	24.39%	26.83%	48.78%
5	2	1	2	3	4	49.23%	24.62%	27.69%
6	2	2	1	4	3	66.32%	21.05%	12.63%
7	2	3	4	1	2	81.00%	5.00%	15.00%
8	2	4	3	2	1	79.38%	9.28%	11.34%
9	3	1	3	4	2	9.85%	31.82%	58.33%
10	3	2	4	3	1	50.50%	37.62%	11.88%
11	3	3	1	2	4	43.53%	25.88%	30.59%
12	3	4	2	1	3	52.17%	30.43%	17.39%
13	4	1	4	2	3	22.03%	33.90%	43.37%
14	4	2	3	1	4	32.69%	33.90%	38.71%
15	4	3	2	4	1	32.26%	33.33%	34.41%
16	4	4	1	3	2	29.07%	32.56%	38.37%

### Induction of adventitious buds after transformation

Intuitive analysis of data (Table 4) showed that the differentiation rates of adventitious buds varied when applying different treatment conditions (different pre-culture time, OD values of the bacterial solution, pH values of the infection solution, infection time and co-culture time). Among them, combination 10 (A3B2C4D3E1) was the best combination, and the differentiation rate of the adventitious rate was 64.36%. The positive rate in fluorescence identification (proportion of fluorescent buds to all adventitious buds) was 54.7% Among the range analysis results, the effects of the five factors on the transformation results were co-culture time (d), infection time (h), concentration of bacterial solution (OD_600_), pre-culture time (d), and pH value of infection solution in descending order. The following combination of buds transformed the quickest: A3B1C4D3E1 with a pre-culture time of 6 d, concentration of bacterial solution (OD_600_) of 0.3, infection solution with pH 5.8, infection time of 2.0 h, and co-culture time of 2 d.

**Table 4:** The results of orthogonal experiments.

Treatment	Column number and effect					Percentage of adventitious buds (%)	Positive rate in fluorescence identification (%)
	1	2	3	4	5		
	A	B	C	D	E		
1	1	1	1	1	1	54.4	42.4
2	1	2	2	2	2	12.15	11.1
3	1	3	3	3	3	42.39	36.8
4	1	4	4	4	4	29.27	27.2
5	2	1	2	3	4	36.92	33.4
6	2	2	1	4	3	38.87	29.1
7	2	3	4	1	2	20.00	18.5
8	2	4	3	2	1	42.27	34.1
9	3	1	3	4	2	26.52	23.7
10	3	2	4	3	1	64.36	54.7
11	3	3	1	2	4	22.35	18.4
12	3	4	2	1	3	47.83	40.3
13	4	1	4	2	3	37.29	31.1
14	4	2	3	1	4	23.08	16.4
15	4	3	2	4	1	21.51	15.2
16	4	4	1	3	2	32.56	26.3
Mean value k_1_	34.575	38.775	37.075	36.33	45.65		
Mean value k_2_	34.525	34.65	29.6	28.55	22.825		
Mean value k_3_	40.275	26.575	33.575	44.075	41.6		
Mean value K_4_	28.625	38	37.75	29.057	27.925		
Range difference R	11.65	12.2	8.15	15.53	22.83		
Adjustment R’	10.485	10.98	7.335	13.98	20.55		

### Analysis of variance for the orthogonal experiments

Analysis of variance (Table 5) revealed that the pre-culture time, OD value of the bacterial solution, pH value of the injection solution, infection time, and co-culture time had significant impact on the differential induction of adventitious buds after genetic transformation; nevertheless, the effects of co-culture time were the greatest.

**Table 5:** Analysis of variance for the effects of different genetic transformation parameters on the differentiation rate of adventitious buds.

No.	Source of variance	Sum of square	Degree of freedom	Mean square	F ratio	*F* critical value	*p* value
1	Pre-culture time (d)	1526.88	3	508.96	65.91	3.290	<0.01
2	Concentration of bacterial solution (OD_600_)	1153.19	3	384.40	49.78	3.290	<0.01
3	Infection solution pH	488.09	3	162.70	21.07	3.290	<0.01
4	Infection time (h)	985.91	3	328.64	42.56	3.290	<0.01
5	Co-culture time (d)	6040.01	3	2013.34	260.72	3.290	<0.01
Error		2872.03	32	7.72			

### Results of fluorescence identification for adventitious buds

**Fig 3:**
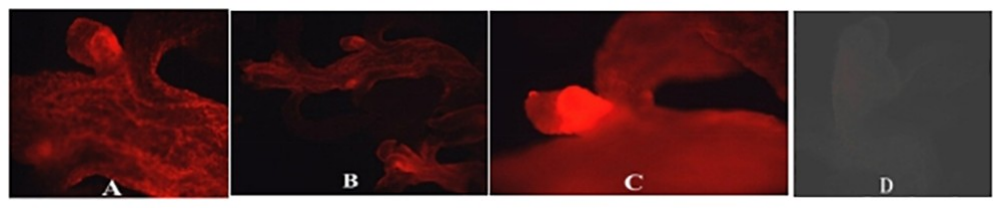
Transformant *Eucalyptus* adventitious buds with *mCherry*. **(A-C**) Transgenic adventitious buds; **D**: Non-transgenic control; Panels A-C show the red fluorescence of the transformant plant tissues and Panel D shows the non-transformed buds.

The induced adventitious buds that grew on the explants were screened using a fluorescence microscope. Significant red fluorescence was visible in some adventitious buds under the excitation light at a wavelength of 580 nm (Fig 3A), while no fluorescence was detected in control adventitious buds that were not transformed (Fig 3D). The percentages of adventitious buds with fluorescence were counted (Table 4).

#### Detection of Cas9 gene in transgenic plants

**Fig 4:**
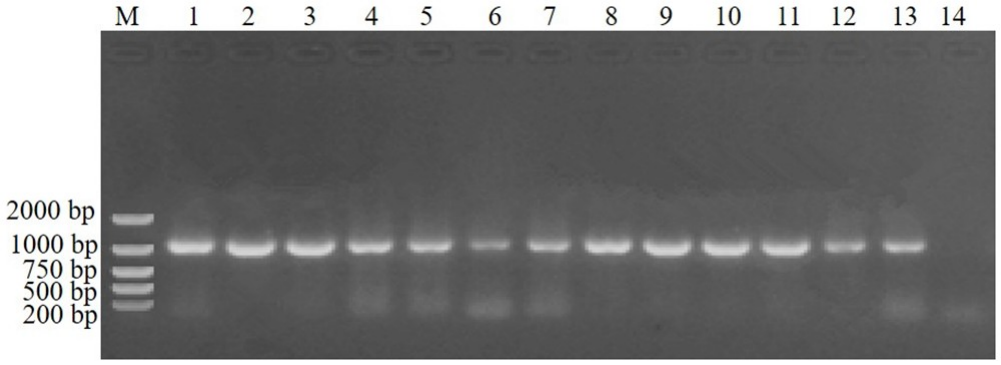
PCR amplification of the Cas9 gene in the plants to be transformed. M: DL2000bp DNA Marker; Electrophoresis lanes 1–12: Plants to be transformed; 13: Template of positive plasmids; 14: Non-transgenic plants.

Amplification results for the Cas9 gene in the plants intended for transformation are shown in Fig 4. The genome of randomly selected plants intended for transformation showed that the Cas9 gene could be amplified to a length of about 1100 bp, but the same segment in non-transgenic plants could not, which is consistent with previous adventitious buds fluorescence results (Fig 3A).

## Discussion

### Factors affecting genetic transformation of *E*. *urophylla × E*. *grandis*

Transgenic technology is a common method for improving the quality of *Eucalyptus*. Nevertheless, the key to a successful *Eucalyptus* transgenesis is to establish a relevant tissue culture and regeneration system, as well as a genetic transformation system. We previously established an efficient regeneration system for *E*. *urophylla × E*. *grandis* [18,19]. So far, only a few studies have examined the genetic transformation system of *E*. *urophylla × E*. *grandis* [4]. Thus, it is very important to establish optimized a genetic transformation system based on the factors affecting the genetic transformation of plant explants.

Numerous factors may affect the genetic transformation rate of plant explants, including plant type and physiological state, explant genotype, genetic transformation methods, type of *Agrobacterium tumefaciens*, culturing conditions, and screening concentration of resistance factors [18]. In addition, the transformation of plant cells using *A*. *tumefaciens* involves a series of complex processes, including the adhesion of *A*. *tumefaciens* to the plant cells; activating the signaling molecules released by the plant cells on the *A*. *tumefaciens* vir gene; and transfer, integrate, and express T-DNA in the nuclear genome of plant cells [20,21]. This study used orthogonal experiments to optimize the following parameters for genetic transformation: pre-culture time, OD value of bacterial solution, pH value of infection solution, infection time, and co-culture time. Moreover, a genetic transformation system in *E*. *urophylla × E*. *grandis* was establishedwith the positive rate of fluorescence identification of up to about 42%.

In our study, the transformation parameters were optimized using orthogonal experiments. Different explants were obtained after 6 d of pre-culture, and embryogenic calli were formed. Meanwhile, *A*. *tumefaciens* easily binds to the dividing cells, improving the transformation efficiency. After 6 d of pre-culture, explant wounds healed and bell-like protrusions on both ends formed, thus reducing the damage from *A*. *tumefaciens* and browning deaths on the explants; this, in turn, facilitated the differentiation of resistant buds. Previous studies have demonstrated that pre-culturing can promote cell division during the *A*. *tumefaciens*-mediated transformation of plant explants by exogenous genes. In addition, it is easier for cells with vigorous division to integrate exogenous DNA, thus improving the transformation rate of exogenous genes [4]. Mezghani et al. [22] observed cell division and tissue differentiation within the wound of the explants 4 d after pre-culture, which is the time when the ability of cells to accept exogenous DNA is the strongest. Furthermore, studies have shown that pre-culture can enhance the tolerance of explants to infection by *A*. *tumefaciens*, and reduce the damage of explants by *A*. *tumefaciens* during the transformation process [23,24].

The effects of time needed for co-culture is also important for the transformation. Co-culture can prevent *A*. *tumefaciens* from affecting explant vitality due to excessive growth, and reduce the degree of damage on the explants due to long-time contacting of *A*. *tumefaciens*. In addition, a suitable amount of co-culture time can also avoid reduction in transformation rate due to insufficient contact between *A*. *tumefaciens* and the explants [11]. In this study, a co-culture was performed for 2 d during the process of genetic transformation of *E*. *urophylla × E*. *grandis*. This was long enough for *A*. *tumefaciens* to fully integrate into the differentiated cells of the explants, which further reduced the percentage of false positive plants, thus improving the transformation rate. Infection time and concentration of bacterial solution also had certain effects on the transformation. Suitable infection time and concentration of bacterial solution can avoid the toxic effects of *A*. *tumefaciens* on the plant cells, and also implement sufficient contact between bacterial solution and the explants, thus increasing the transformation rate. In the present study, the induction rate of resistant adventitious buds was highest after 2 h of infection when the concentration of the *A*. *tumefaciens* solution (OD_600_) was 0.3.

### Application of screening marker genes in the efficient transformation system of *Eucalyptus*

Successful A. tumefaciens- mediated transformation in most plants depends on the effectiveness of appropriate selection conditions. The antibiotic is widely used as a selectable marker; therefore, its sensitivity should be determined at the initial stages of the development of a plant transformation system in which a antibiotic resistance gene is used [25]. But antibiotic screening is easy to produce false positive. So, antibiotic screening is less efficient. *mCherry* is a monomeric red fluorescent protein that absorbs light between 540–590 nm and emits light at 550–650 nm. *mCherry* is tolerant to photobleaching, and its fluorescence is very stable. *mCherry* is usually used to label and trace molecules and cellular components. Compared to other fluorescent proteins, *mCherry* has excellent photostability, which is why it has been extensively applied in biological studies and preclinical work [26]. In this study, we cloned the *mCherry* close to promoter 35S (a strong promoter commonly used in plant transformation) to examine the gene transformation efficiency.

Our data revealed different positive rates of fluorescence identification under different transformation conditions, and the positive rate showed a positive linear relationship with the percentage of resistance buds. Studies have shown that the occurrence of embryoid bodies is usually caused by a single cell. Therefore, fewer chimeras are obtained through transformation in the transgenic plants. So, selectable marker *mCherry* screening is efficient. There is no previous article on this method of screening markers. This system can be used as a plant genome site-specific editing tool and may be useful for improving *Eucalyptus* genetic resources. The transformation rate can greatly improve, and the percentage of chimeras can be reduced if an embryogenic transformation system is established. Regenerated plants can be obtained via the regeneration way of the embryoid bodies in future studies.

## Supporting information

S1 Raw images (PDF)
