# Supplementary figures and images for: Efficient genetic transformation method for Eucalyptus genome editing
Source: PLoS One. 2021 May 24;16(5):e0252011. doi: 10.1371/journal.pone.0252011 (PMC8143419; doi:10.1371/journal.pone.0252011)

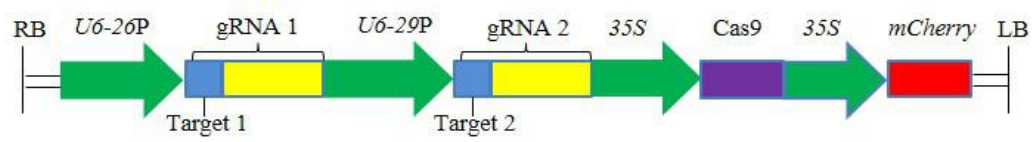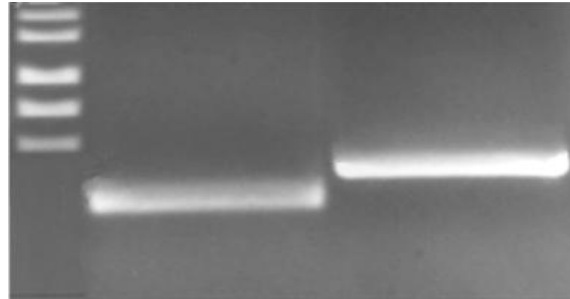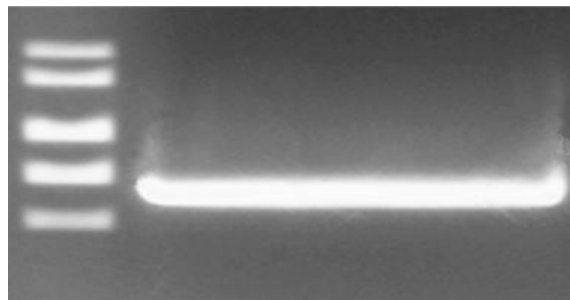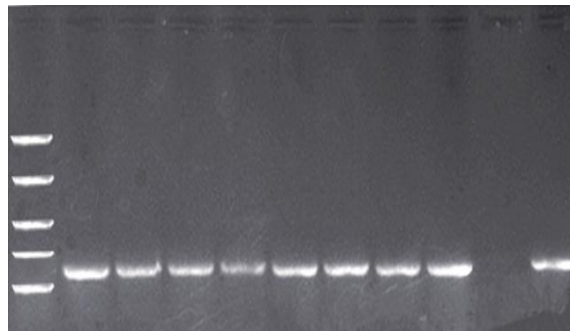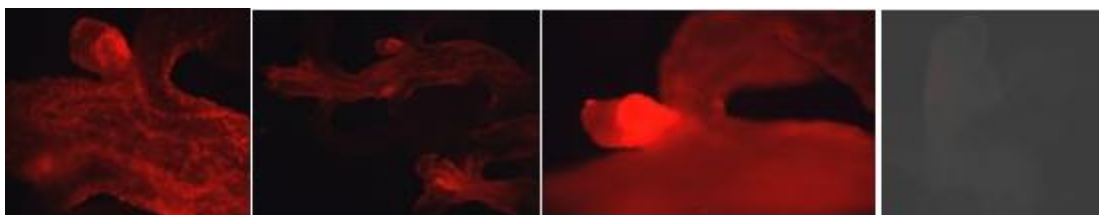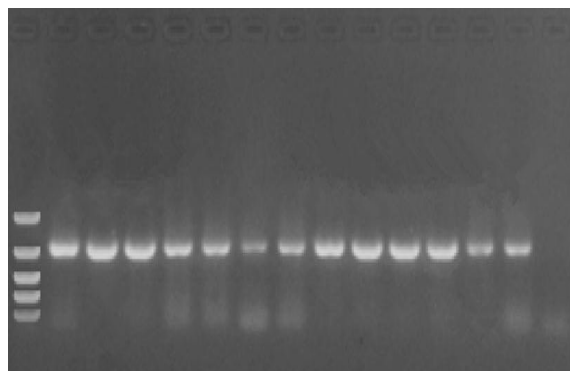

Supplement: S1 Raw images — (PDF) [file pone.0252011.s001.pdf]
